# Flexible Nets: a modeling formalism for dynamic systems with uncertain parameters

**DOI:** 10.1007/s10626-019-00287-9

**Published:** 2019-08-22

**Authors:** Jorge Júlvez, Stephen G. Oliver

**Affiliations:** 1 Cambridge Systems Biology Centre, University of Cambridge, Cambridge, UK; 2 Department of Biochemistry, University of Cambridge, Cambridge, UK; 3 Department of Computer Science and Systems Engineering, University of Zaragoza, Zaragoza, Spain

**Keywords:** Flexible nets, Modeling formalisms, Petri nets, Dynamic systems, Uncertain parameters, Performance analysis

## Abstract

The modeling of dynamic systems is frequently hampered by a limited knowledge of the system to be modeled and by the difficulty of acquiring accurate data. This often results in a number of uncertain system parameters that are hard to incorporate into a mathematical model. Thus, there is a need for modeling formalisms that can accommodate all available data, even if uncertain, in order to employ them and build useful models. This paper shows how the Flexible Nets (FNs) formalism can be exploited to handle uncertain parameters while offering attractive analysis possibilities. FNs are composed of two nets, an event net and an intensity net, that model the relation between the state and the processes of the system. While the event net captures how the state of the system is updated by the processes in the system, the intensity net models how the speed of such processes is determined by the state of the system. Uncertain parameters are accounted for by sets of inequalities associated with both the event net and the intensity net. FNs are not only demonstrated to be a valuable formalism to cope with system uncertainties, but also to be capable of modeling different system features, such as resource allocation and control actions, in a facile manner.

## Introduction

The development of appropriate models is crucial for the design, analysis and control of dynamic systems. The usefulness of a model depends on both its capacity to capture the relevant features of the system, and its capacity for mathematical analysis. These capacities of the model largely rely on the adopted modeling formalism, i.e. on the set of modeling principles and rules that are used to build the model. The task of modeling is often hindered by the lack of detailed system information.

This paper exploits the particular features of Flexible Nets (FNs), a modeling formalism introduced in Júlvez et al. (2018) to study Wilson disease, to model and analyze dynamic systems with uncertain parameters, to account for partially observable systems and, to compute the control actions that optimize a given control objective. Roughly speaking, a dynamic system can be seen as a set of *state* variables that are modified by means of *processes* (by *process*, we mean any event, operation or activity whose occurrence has the potential to change the state of the system). These two basic entities, *state* and *process*, are mutually related: on the one hand, the execution of the processes determines how the state changes; on the other hand, the state determines the speed of the processes. These relationships between state and processes are clear, for instance, in a chemical system where the state is given by the amount of molecules and the processes are the reactions taking place in the system. On the one hand, the occurrence of reactions produces a change in the amount of molecules that satisfies the stoichiometry of these reactions. On the other hand, the rate of the reactions depends on the amount of molecules. FNs capture these relationships between state and processes by means of two different nets: the event net and the intensity net.

Let us introduce some of the basic features of FNs by means of a simple chemical reaction network. Assume that the reaction network is composed of the following two reactions:

$$R_{1}:\emptyset \rightarrow\ A\hspace{3cm} R_{2}: A\rightarrow\ nB+4nC$$

Reaction *R*_1_ models the production of compound *A* (each occurrence of *R*_1_ increases the concentration of *A*, which is denoted [*A*], one unit), and reaction *R*_2_ models how *A* is decomposed into compounds *B* and *C*. The amounts which [*B*] and [*C*] are increased by the occurrence of *R*_2_ depend on *n*. Let us assume that *n* is uncertain, but known to be in the interval [20,22]. That is, each occurrence of *R*_2_ decreases [*A*] one unit, increases [*B*] *n* units, and increases [*C*] 4*n* units where *n* ∈ [20,22]. Let us further assume that the initial concentrations of [*B*] and [*C*] are 0, and the initial concentration of [*A*] is known to be in the interval [9.9,1.1].

**Fig. 1:**
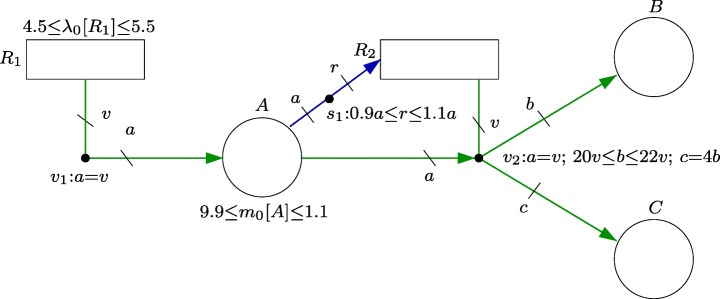
FN modeling a simple chemical reaction network

The FN in Fig. 1 models the described reaction network. Namely, each chemical compound is a associated with a circle (which will be called a place), and each reaction is associated with a rectangle (which will be called a transition). The stoichiometry of the reactions is modeled by the equations associated with the dots labelled *v*_1_ and *v*_2_ (which will be called event handlers). The uncertain stoichiometry of *R*_2_ is accounted for by the inequalities associated with *v*_2_, i.e. *a*=*v*, 20*v*≤*b*≤ 22*v* and *c*= 4*b*; and the uncertain initial concentration [*A*] is captured by the inequalities associated with *A*, i.e. 9.9≤*m*_0_[*A*]≤ 1.1.

The event net of an FN is a graph with three different types of vertices: *places*, *transitions* and *event handlers*. While places and transitions are used to model the state and the processes of the system, respectively, event handlers are used to determine the quantities by which the state is changed when given processes occur. Each place is associated with a state variable, and the value of that variable at a given instant is called marking, or number of *tokens*, in that place. Similarly, each transition is associated with a process, and the number of times the process has taken place is the number of *actions* in the transition. Each event handler connects a set of transitions to a set of places, and is associated with a set of linear inequalities that relates actions to marking. Given a number of actions in the connected transitions, any solution of the set of linear inequalities can be used to update the number of tokens in the connected places. Thus, the amount by which the marking changes is allowed to be nondeterministic. This feature of event nets allows the model to account for the different system evolutions that can arise as a consequence of uncertainty in the system. In Fig. 1, the event net is composed of the places, transitions, event handlers and arcs and edges in green.

Let us further assume that the rate of reaction *R*_1_ is uncertain, but constrained to the interval [4.5,5.5], i.e. the number of reactions that occur per time unit is in [4.5,5.5], and the rate *R*_2_ satisfies 0.9[*A*] ≤ *rate*(*R*_2_) ≤ 1.1[*A*], i.e. it is proportional to [*A*] with an uncertainty of 10*%*. These reaction rates are modeled in the FN by the inequality associated with *R*_1_ and the inequality associated with the dot labelled *s*_1_ (which will be denoted intensity handler).

Similarly to event nets, an intensity net is a graph with three different types of vertices: *places*, *transitions* and *intensity handlers*. Places and transitions have the same role as in event nets. Each intensity handler connects a set of places to a set of transitions, and is associated with a set of linear inequalities that relates the number of tokens with the speed of the transitions, i.e. of the processes modeled by the transitions. The intensity, or speed, of a transition determines the rate at which actions are created in the transition. As in event nets, any solution of the inequalities can be used to determine the speed of transition, thus, linear inequalities can be used to model uncertainties in the dynamics of the system. In Fig. 1, the intensity net is composed by the places, transitions, intensity handlers and arcs and edges in blue.

An FN is the result of combining an event net and an intensity net, i.e. an FN is a graph with four types of vertices: *places*, *transitions*, *event handlers* and *intensity handlers*. While the intensity net establishes the speeds at which actions are generated as a function of the marking, the event net specifies how the generated actions are executed and how a new marking is computed. Thus, although FNs are inspired by Petri nets (Murata 1989), their structure is different, since in addition to *places* and *transitions*, FNs have *handlers* which are connected to places and transitions by *arcs* and *edges*. FNs offer both high modeling power and appealing analysis possibilities that aim to make use of all the information provided by the available uncertain parameters. Namely, FNs can accommodate uncertainties in the initial marking, in the marking change produced by the firing of the transitions, in the default speeds of transitions, and in the speed of transitions produced by the marking.

A number of different formalisms can be found in the literature that can, to some extend, incorporate uncertain parameters in their models. Depending on the domain of their state variables, these formalisms can be roughly classified as those whose variables are integer numbers, and those whose variables are real numbers (hybrid approaches combine both types of variables).

In the discrete domain, extensions of the most popular modeling formalisms exist that can handle uncertain parameters. For instance, in the Petri nets arena, uncertain knowledge of the marking is particularly well handled by fuzzy (Looney 1988) and possibilistic Petri nets (Cardoso et al. 1999), uncertainty in the firing of transitions can be accounted for by labeled (Cabasino et al. 2010) and interpreted (Ramirez-Trevino et al. 2003) Petri nets, and uncertain firing times can be modeled by time Petri nets (Merlin and Faber 1976) and stochastic Petri nets (Ajmone Marsan et al. 1995). Other discrete formalisms that include extensions to account for uncertain parameters are probabilistic Boolean networks (Shmulevich et al. 2002), which are an extension of Boolean networks (Wang et al. 2012), and influence diagrams (Detwarasiti and Shachter 2005), which are generalizations of Bayesian networks (Needham et al. 2007) that can solve decision problems under uncertainty. Stochastic extensions of timed automata (Alur and Dill 1994) also exist in which delays and discrete choices are made randomly (Bertrand et al. 2014). In a similar vein, stochastic extensions of process algebras (Priami et al. 2001; Ciocchetta and Hillston 2008) have been proposed to describe components with uncertain behaviour (Clark et al. 2007).

A major difference of the above mentioned approaches with respect to FNs is that the state variables of FNs are real numbers. This implies that genuinely discrete systems cannot be modeled by FNs. Nevertheless, the use of real variables facilitates, in general, the use of more efficient analysis methods since the state explosion problem inherent to large discrete systems is avoided, and linear programming techniques can be applied. Moreover, large discrete populations can be approximated reasonably well in many cases by means of real variables (Bortolussi et al. 2013). With respect to the existing stochastic approaches and extensions, it should be said that they offer the possibility to perform useful statistical analyses, which usually require information about the probability distributions of the system, and often involve a significant computational cost. In contrast, FNs do not require information about probability distributions, just about the intervals in which the uncertain parameters lay. This results in efficient analysis techniques based on linear programming.

The most popular modeling approaches in the continuous domain are based on differential equations (Braun et al. 1983; Tyson et al. 2011; van den Berg et al. 2008). In particular, the relaxation of the integrality constraint in a discrete formalism usually leads to models that are governed by differential equations. For instance, the evolution of continuous Petri nets (Silva et al. 2011), which can be seen as a relaxation of Petri nets (Murata 1989), is determined by a set of ordinary differential equations. Another popular modeling formalism that can graphically represent systems in different domains and that can be easily converted to state space representation is bond graphs (Borutzky 2010). However, it should be emphasised that a potential difficulty in the design of models based on differential equations is that exhaustive and accurate information about the system dynamics is required, i.e. uncertain parameters cannot be easily handled. Note that the time trajectory of a system modeled by ordinary differential equations is continuous and deterministic. On the other hand, constraint-based models, which are popular in systems biology (Varma and Palsson 1994; Orth et al. 2011), can incorporate uncertain dynamic information but their analysis capabilities are limited to the steady state.

An important feature of FNs is that they can bridge the gap between deterministic models and constraint-based models by allowing the incorporation of uncertain parameters. Thus, on the one hand and similarly to continuous Petri nets which are deterministic (Jiménez et al. 2004), FNs can model positive linear systems; on the other hand and similarly to constraint-based models (Varma and Palsson 1994), FNs can model systems with uncertain initial state and uncertain process speeds. In contrast to Petri nets, the timing of transitions and the marking changes in places are explicitly separated in FNs: the timing is handled by the intensity handlers, and the marking changes by the event handlers. This can lead to a clearer and more concise graphical representation of the system. Moreover, efficient computational methods exist to analyse the transient state of FNs.

The rest of the paper is organized as follows: Section 2 introduces event nets and shows how a partially observable system can be modeled. Intensity nets are presented in Section 3. The combination of these nets leads to FNs, which are defined in Section 4. Section 5 shows how FNs can handle systems with uncertain parameters and analyze them. Section 6 concludes the paper.

## Event nets

In the following, the reader is assumed to be familiar with Petri nets (see Murata^1989^ for a gentle introduction).

### Definition and state equations

This section introduces event nets, which can be denoted as *TVP* nets, i.e. actions in transitions *T* produce and consume tokens in places *P* through event handlers *V*. Event handlers connect places and transitions, and determine the marking changes according to the actions in the transitions. In contrast to Petri nets, the net elements that produce changes in the marking are the event handlers, and such changes are allowed to be nondeterministic.

#### **Definition 1** (Event net)

An event net is a tuple ${{\mathcal {N}}}_{V}=(P,T,V,E_{V},A,B)$ where (*P*, *T*, *V*, *E*_*V*_) is a tripartite graph determining the net structure and (*A*, *B*) are matrices determining the potential evolutions of the marking.

The set of vertices of the net is partitioned into three sets:

- *P* = {*p*_1_,…,*p*_*i*_,…} is a set of |*P*| places.
- *T* = {*t*_1_,…,*t*_*j*_,…} is a set of |*T*| transitions.
- *V* = {*v*_1_,…,*v*_*k*_,…} is a set of |*V* | event handlers.

The places, depicted as circles, model the different types of components or elements in the system, e.g. resources, products, items, etc. The transitions, depicted as rectangles, model the different types of operations, activities or processes in the system. Such operations require time to be performed and have the potential to change, i.e. produce and consume, the amount of components, i.e. the marking. The event handlers, depicted as dots, model the different ways in which the transitions can change the marking.

The vertices of the net are connected by the edges in *E*_*V*_. Each pair of vertices can be connected by at most one edge. The set *E*_*V*_ is partitioned into two sets ${E_{V}^{P}}$ and ${E_{V}^{T}}$, where ${E_{V}^{P}}$ is a set of *directed edges* connecting places to event handlers and vice versa, and ${E_{V}^{T}}$ is a set of *undirected edges* connecting transitions and event handlers. For simplicity, directed edges are referred as *arcs*, and undirected edges as *edges*. More formally:

- Every $e\in {E_{V}^{P}}$ is either an arc *e* = (*p*_*i*_,*v*_*k*_) from a place *p*_*i*_ to a handler *v*_*k*_, or an arc *e* = (*v*_*k*_,*p*_*i*_) from a handler *v*_*k*_ to a place *p*_*i*_.
- Every $e\in {E_{V}^{T}}$ is an edge *e* = {*t*_*j*_,*v*_*k*_} connecting a transition *t*_*j*_ and a handler *v*_*k*_.

Notice that direct connections among places and transitions are not allowed. The following notation is used:

- ^*p*^*v*_*k*_ denotes the input places of *v*_*k*_, i.e. $^{p}v_{k}=\{p_{i}|(p_{i},v_{k})\in {E_{V}^{P}}\}$
- ${v_{k}^{p}}$ denotes the output places of *v*_*k*_, i.e. ${v_{k}^{p}}=\{p_{i}|(v_{k},p_{i})\in {E_{V}^{P}}\}$
- ^*v*^*p*_*i*_ denotes the input handlers of *p*_*i*_, i.e. $^{v}p_{i}=\{v_{k}|(v_{k},p_{i})\in {E_{V}^{P}}\}$
- ${p_{i}^{v}}$ denotes the output handlers of *p*_*i*_, i.e. ${p_{i}^{v}}=\{v_{k}|(p_{i},v_{k})\in {E_{V}^{P}}\}$
- ^*t*^*v*_*k*_ denotes the transitions connected to *v*_*k*_, i.e. $^{t}v_{k}=\{t_{j}|\{t_{j},v_{k}\}\in {E_{V}^{T}}\}$
- ${t_{j}^{v}}$ denotes the handlers connected to *t*_*j*_, i.e. ${t_{j}^{v}}=\{v_{k}|\{t_{j},v_{k}\}\in {E_{V}^{T}}\}$

#### *Example 1*

**Fig. 2:**
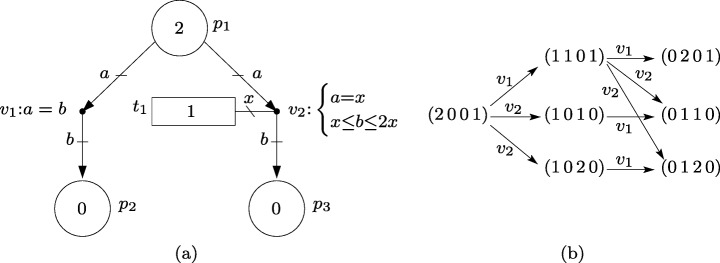
**a** Event net. **b** Potential state evolutions with discrete firings of the event handlers (the components of the vectors correspond to the variables (*m*[*p*_1_] *m*[*p*_2_] *m*[*p*_3_] *a*_*T*_[*t*_1_]))

The event net in Fig. 2a has three places, *P* = {*p*_1_,*p*_2_,*p*_3_}, one transition, *T* = {*t*_1_}, and two event handlers *V* = {*v*_1_,*v*_2_}. The set of arcs is ${E_{V}^{P}}=\{(p_{1},v_{1}), (v_{1},p_{2}), (p_{1}, v_{2}), (v_{2},p_{3})\}$, and the set of edges is ${E_{V}^{T}}=\{\{t_{1},v_{2}\}\}$. The set of all arcs and edges is $E_{V}={E_{V}^{P}}\cup {E_{V}^{T}}$. As examples for the introduced notation, the set of output handlers of *p*_1_ is ${p_{1}^{v}}=\{v_{1},v_{2}\}$, the set of transitions connected to *v*_2_ is ^*t*^*v*_2_ = {*t*_1_}, and the set of input handlers of *p*_3_ is ^*v*^*p*_3_ = {*v*_2_}.

In an event net, each place contains a number of tokens (or marking), and each transition contains a number of actions that represent the potential of the system to carry out the associated process. In contrast to tokens, actions require time to be produced (the production rate of actions is determined by the intensity net, see Section 3). The state of an event net accounts not only for the marking and the number of actions, but also for the marking changes and the execution of actions:

#### **Definition 2** (State)

The state of an event net ${{\mathcal {N}}}_{V}$ is given by the tuple (*σ*, *a*_*T*_,*a*_*E*_,Δ*m*, *m*), where:

- $\sigma \in \mathbb {R}^{|T|}_{\geq 0}$ is a vector indexed by *T* where *σ*[*t*_*j*_] is the number of actions produced in *t*_*j*_.
- $a_{T}\in \mathbb {R}^{|T|}_{\geq 0}$ is a vector indexed by *T* where *a*_*T*_[*t*_*j*_] is the number of actions available in *t*_*j*_.
- $a_{E}\in \mathbb {R}^{|{E_{V}^{T}}|}_{\geq 0}$ is a vector indexed by ${E_{V}^{T}}$ where *a*_*E*_[{*t*_*j*_,*v*_*k*_}] is the number of actions of *t*_*j*_ executed by *v*_*k*_.
- ${\Delta }m\in \mathbb {R}^{|{E_{V}^{P}}|}_{\geq 0}$ is a vector indexed by ${E_{V}^{P}}$ where Δ*m*[(*p*_*i*_,*v*_*k*_)] is the number of tokens in *p*_*i*_ consumed by *v*_*k*_, and Δ*m*[(*v*_*k*_,*p*_*i*_)] is the number of tokens in *p*_*i*_ produced by *v*_*k*_.
- $m\in \mathbb {R}^{|P|}_{\geq 0}$ is the marking, i.e. a vector indexed by *P* where *m*[*p*_*i*_] is the number of tokens in *p*_*i*_.

Thus, every net element (except for the event handlers) is associated with at least one nonnegative real variable. Since actions need time to be produced, at the initial state it holds *σ* = 0, *a*_*T*_ = 0 and *a*_*E*_ = 0. Notice that dealing with real state variables instead of discrete ones allows the model to incorporate real quantities and to approximate large discrete quantities as in continuous Petri nets (Silva et al. 2011). In any case, the state variables can be constrained to the nonnegative integers if required, see Section 2.2.

Each event handler *v*_*k*_ ∈ *V* is associated with a set of linear inequalities that relate the number of actions executed in the connected transitions to the marking changes in the connected places. The coefficients of such a set of inequalities can be expressed by two matrices (*A*_*k*_,*B*_*k*_) of real numbers and the same number of rows that are associated with each handler *v*_*k*_ ∈ *V*. The number of actions executed by *v*_*k*_, *a*_*f*_, and the produced marking changes, Δ*m*_*f*_, is given by *A*_*k*_Δ*m*_*f*_≤*B*_*k*_*a*_*f*_. The columns of *A*_*k*_ are indexed by the arcs connecting *v*_*k*_ to places. The columns of *B*_*k*_ are indexed by the edges connecting transitions to *v*_*k*_. Matrix *A*(*B*) is obtained by arranging all the matrices *A*_*k*_(*B*_*k*_) *diagonally*.

#### *Example 2*

The inequalities associated with the event handlers of the net in Fig. 2a are: *v*_1_:*a*=*b* and $v_{2}{:}\left \{\begin {array}{cc}a{=}x\\ x{\leq } b{\leq } 2x \end {array}\right .$, where ’*a*’, ’*b*’ and ’*x*’ are used to label arcs and edges. More precisely, in *v*_1_:*a*=*b*, ’*a*’ denotes the number of tokens in *p*_1_ consumed by *v*_1_, and ’*b*’ denotes the number of tokens in *p*_2_ produced by *v*_1_. In the inequalities associated with *v*_2_, ’*a*’ and ’*b*’ denote the number of tokens in *p*_1_ consumed by *v*_2_ and the number of tokens in *p*_3_ in produced by *v*_2_ respectively, and ’*x*’ denotes the number of actions in *t*_1_ executed by *v*_2_. The equality *v*_1_:*a*=*b* means that the number of tokens in *p*_1_ consumed by *v*_1_ is equal to the number of tokens in *p*_2_ produced by *v*_1_. In other words, for every token in *p*_1_ consumed by *v*_1_, a token is produced in *p*_2_ by *v*_1_, this can be interpreted as tokens moving from *p*_1_ to *p*_2_ through *v*_1_.

The equation *a*=*x* associated with *v*_2_ means that the number of tokens in *p*_1_ consumed by *v*_2_ is equal to the number of actions in *t*_1_ executed by *v*_2_, e.g. if one action is executed then one token is consumed. Moreover, the inequality *x*≤*b*≤ 2*x* means that the execution of one action in *t*_1_ by *v*_2_, i.e. *x* = 1, produces a nondeterministic quantity *b* ∈ [1,2] of tokens in *p*_3_ (each execution of an action can produce a different amount *b* ∈ [1,2] of tokens in *p*_3_).

Notice that *v*_1_ is not connected to any transition. This means that no process is required to move a token from *p*_1_ to *p*_2_. For the sake of mathematical notation, it can be assumed that *v*_1_ is connected to a fake transition, *t*_*fake*_, that has no effect on the model. The matrices *A*_1_, *B*_1_, *A*_2_ and *B*_2_ that capture the inequalities associated with the event handlers are:

$$ A_{1}{=} \left( \begin{array}{cc} 1 & -1\\ -1 & 1 \end{array}\right) ; B_{1}{=} \left( \begin{array}{l} 0\\ 0 \end{array}\right) ; A_{2}{=} \left( \begin{array}{cccc} 1 & 0\\ -1 & 0\\ 0 & -1\\ 0 & 1 \end{array}\right) ; B_{2}{=} \left( \begin{array}{ll} 1\\ -1\\ -1\\ 2 \end{array}\right) $$

where the indices of the columns of *A*_1_ are ordered as (*p*_1_,*v*_1_), (*v*_1_,*p*_2_); the index of the column of *B*_1_ is {*t*_*fake*_,*v*_1_}; the indices of the columns of *A*_2_ are ordered as (*p*_1_,*v*_2_), (*v*_2_,*p*_3_); and the index of *B*_2_ is {*t*_1_,*v*_2_}. Thus, the number of actions executed and marking changes produced by *v*_2_ are related by:

$$ \left( \begin{array}{cc} 1 & 0\\ -1 & 0\\ 0 & -1\\ 0 & 1 \end{array}\right) \left( \begin{array}{cc} {\Delta}m[(p_{1},v_{2})]\\ {\Delta}m[(v_{2},p_{3})] \end{array}\right) {\leq} \left( \begin{array}{cc} 1\\ -1\\ -1\\ 2 \end{array}\right) \left( \begin{array}{cc} a_{E}[\{t_{1},v_{2}\}] \end{array}\right) $$

Matrices *A*_1_ and *A*_2_(*B*_1_ and *B*_2_) can be arranged diagonally to obtain *A*(*B*):

$$ A= \left( \begin{array}{cccc} 1 & -1 & 0 & 0\\ -1 & 1 & 0 & 0\\ 0 & 0 & 1 & 0\\ 0 & 0 & -1 & 0\\ 0 & 0 & 0 & -1\\ 0 & 0 & 0 & 1 \end{array}\right) ;\ \ B= \left( \begin{array}{cc} 0 & 0 \\ 0 & 0 \\ 0 & 1 \\ 0 & -1 \\ 0 & -1 \\ 0 & 2 \end{array}\right) $$

where the indices of the columns of *A* are (*p*_1_,*v*_1_), (*v*_1_,*p*_2_), (*p*_1_,*v*_2_), (*v*_2_,*p*_3_); and the indices of the columns of *B* are {*t*_*fake*_,*v*_1_}, {*t*_1_,*v*_2_}.

Notice that the number of actions produced in a transition (by the intensity net), *σ*, is equal to the number of actions that have been executed by the connected event handlers, *a*_*E*_, plus the number of actions still available, *a*_*T*_, hence, it holds:

1 $$  \sigma[t_{j}]=a_{T}[t_{j}]+\sum\limits_{v_{k}\in {t_{j}^{v}}} a_{E}[\{t_{j},v_{k}\}]\ \ \forall\ t_{j}\in T $$

Similarly, the number of tokens in a place *p*_*i*_ is equal to the initial number of tokens, which is denoted *m*_0_[*p*_*i*_], minus the number of tokens consumed plus the number of tokens produced by the connected event handlers:

2 $$  m[p_{i}]{=}m_0[p_{i}] {-} \sum\limits_{v_{k}\in {p_{i}^{v}}} {\Delta}m[(p_{i},v_{k})] {+}{\sum}_{v_{k}\in^{v}p_{i}} {\Delta}m[(v_{k},p_{i})]\ \ \forall\ p_{i}\in P $$

The event net establishes how the state evolves as event handlers are enabled and fire.

#### **Definition 3** (Enabling)

Event handler *v*_*k*_ is enabled at (*σ*, *a*_*T*_,*a*_*E*_,Δ*m*, *m*) if a vector $a_{f}\in \mathbb {R}_{\geq 0}^{|^{t}v_{k}|}$ indexed by the edges of *v*_*k*_, and a vector ${\Delta }m_{f}\in \mathbb {R}_{\geq 0}^{|^{p}v_{k}|+|{v_{k}^{p}}|}$ indexed by the arcs of *v*_*k*_ exist such that:

3 $$ \begin{array}{@{}rcl@{}} a_{f}[\{t_{j},v_{k}\}] &\leq& a_{T}[t_{j}]\ \ \ \forall\ t_{j}\in ^{t}v_{k} \end{array} $$

4 $$ \begin{array}{@{}rcl@{}} A_{k} {\Delta}m_{f} &\leq& B_{k} a_{f} \end{array} $$

5 $$ \begin{array}{@{}rcl@{}} {\Delta}m_{f}[(p_{i},v_{k})] &\leq& m[p_{i}]\ \ \  \forall\ p_{i}\in ^{p}v_{k} \end{array} $$

6 $$ \begin{array}{@{}rcl@{}} \mathbf{1}a_{f} + \mathbf{1}{\Delta}m_{f} & >& 0 \end{array} $$

Inequality (3) guarantees that enough actions are available, (4) makes use of the matrices *A*_*k*_ and *B*_*k*_ to relate the number of executed actions to the marking changes, (5) guarantees that enough tokens are available in the input places to be consumed, (6) guarantees that the overall state change is not null. Notice that the inequalities (4) allow the modeling of uncertainty in the marking changes produced by the execution of actions.

#### **Definition 4** (Firing)

An event handler *v*_*k*_ enabled at (*σ*, *a*_*T*_,*a*_*E*_,Δ*m*, *m*) can fire. The firing of *v*_*k*_ leads instantaneously to a new state $(\sigma ,a_{T}^{\prime },a_{E}^{\prime },{\Delta }m^{\prime },m^{\prime })$ where only the variables associated with edges, arcs, places and transitions connected to *v*_*k*_ are updated as follows:

$$ \begin{array}{@{}rcl@{}} a_{T}^{\prime}[t_{j}] & =& a_{T}[t_{j}] - a_{f}[\{t_{j},v_{k}\}]~~~~~~~~~~~~~~~~~~~~~~~~~~~~~~~~~~ \forall \ t_{j}{\in} ^{t}v_{k}\\ a_{E}^{\prime}[\{t_{j},v_{k}\}] &=& a_{E}[\{t_{j},v_{k}\}] + a_{f}[\{t_{j},v_{k}\}]~~~~~~~~~~~~~~~~~~~~~~~~~ \forall \ t_{j}{\in} ^{t}v_{k}\\ {\Delta}m^{\prime}[(p_{i}, v_{k})] & =& {\Delta}m[(p_{i}, v_{k})] + {\Delta}m_{f}[(p_{i}, v_{k})]~~~~~~~~~~~~~~~~~ \forall \ p_{i}{\in} ^{p}v_{k}\\ {\Delta}m^{\prime}[(v_{k}, p_{i})] & =& {\Delta}m[(v_{k}, p_{i})] + {\Delta}m_{f}[(v_{k}, p_{i})]~~~~~~~~~~~~~~~~~ \forall \ p_{i}{\in} {v_{k}^{p}}\\ m^{\prime}[p_{i}]&=&m[p_{i}]{-}{\Delta}m_{f}[(p_{i}, v_{k})]~~~~~~~~~~~~~~~~~~~~~~~~~~~~~~~ \forall \ p_{i}{\in} ^{p}v_{k}\\ m^{\prime}[p_{i}]&=&m[p_{i}]{+} {\Delta}m_{f}[(v_{k}, p_{i})]~~~~~~~~~~~~~~~~~~~~~~~~~~~~~~~ \forall \ p_{i}{\in} {v_{k}^{p}} \end{array} $$

where *a*_*f*_ and Δ*m*_*f*_ satisfy (3), (4), (5) and (6).

Notice that an enabled handler is not forced to fire, and that the state reached by the firing of an event handler is allowed to be nondeterministic (see inequality (4)). Moreover, the firing does not force the execution of a minimum number of actions nor the consumption or production of a minimum number of tokens. In fact, the equations in Definition 4 are trivially satisfied with *a*_*f*_ = 0 and Δ*m*_*f*_ = 0. Thus, such equations also hold for every non-enabled handler with *a*_*f*_ = 0 and Δ*m*_*f*_ = 0.

The overall change in the state produced by several firings is the result of adding the changes produced by each firing. This leads to a set of equations that are satisfied by the states that can be reached from the initial state.

#### **Proposition 1** (State equations)

Let the state of an event net ${{\mathcal {N}}}_{V}$ be (*σ*, *σ*,0,0,*m*_0_), i.e. *σ* actions are available and no event handler has fired. Every state (*σ*, *a*_*T*_,*a*_*E*_,Δ*m*, *m*) reachable from (*σ*, *σ*,0,0,*m*_0_) belongs to $SE_{{{\mathcal {N}}}_{V}}(\sigma ,m_0)$ where:

7 $$ \begin{array}{ll} SE_{{{\mathcal{N}}}_{V}}(\sigma,m_0) = \{(&\sigma,a_{T},a_{E},{\Delta}m,m)| \\ & \sigma = a_{T} + Y_{\sigma} a_{E}\\ & A {\Delta}m \leq B a_{E}\\ & m = m_0 + Z_{m} {\Delta}m\} \end{array} $$

where *Y*_*σ*_ and *Z*_*m*_ are determined by the net structure:

- *Y*_*σ*_ is a matrix with rows indexed by *T*, columns indexed by ${E_{V}^{T}}$, and such that $Y_{\sigma }[t_{j},\{t_{j},v_{k}\}]=1\ \forall \ \{t_{j},v_{k}\}\in {E_{V}^{T}}$ and the rest of the elements in *Y*_*σ*_ are 0,
- *Z*_*m*_ is a matrix with rows indexed by *P*, columns indexed by ${E_{V}^{P}}$, and such that $Z_{m}[p_{i},(p_{i},v_{k})]=-1\ \forall \ (p_{i},v_{k}) \in {E_{V}^{P}}$, $Z_{m}[p_{i},(v_{k},p_{i})]=1\ \forall \ (v_{k},p_{i}) \in {E_{V}^{P}}$ and the rest of the elements in *Z*_*m*_ are 0,

and *a*_*T*_, *a*_*E*_, Δ*m* and *m* are nonnegative variables.

#### *Proof*

Let us show that equations (7) necessarily hold for every state (*σ*, *a*_*T*_,*a*_*E*_,Δ*m*, *m*) reachable from (*σ*, *σ*,0,0,*m*_0_). Equation *σ* = *a*_*T*_ + *Y*_*σ*_*a*_*E*_ in (7) states that the number of actions produced, *σ*, is equal to the number of actions executed, *a*_*E*_, plus the number of actions available, *a*_*T*_. This is equivalent to (1) expressed in matrix form, and thus necessarily holds. Equation *A*Δ*m* ≤ *Ba*_*E*_ in (7) is the matrix form of (4) and accounts for all the actions executed and all the marking changes produced by the firing of all the event handlers. That is, it captures all the cumulative marking changes, Δ*m*, produced by all the executed actions, *a*_*E*_, and, hence, must necessarily hold. Finally, equation *m* = *m*_0_ + *Z*_*m*_Δ*m* in (7) which updates the number of tokens, *m*, in all the places according to the cumulative marking changes, Δ*m*, is the matrix form of (2) and, hence, must also hold. □

Roughly speaking, the role of matrix *Y*_*σ*_ is to distribute the actions in transitions among the handlers connected to them, see (1). The role of *Z*_*m*_ is to collect and add the marking changes produced by the firings, see (2).

#### *Example 3*

Equation *σ* = *a*_*T*_ + *Y*_*σ*_*a*_*E*_ for the event net in Fig. 2a assuming again that *v*_1_ is connected to a fake transition is:

$$ \left( \begin{array}{cc} \sigma[t_{1}]\\ \sigma[t_{fake}] \end{array}\right) = \left( \begin{array}{cc} a_{T}[t_{1}]\\ a_{T}[t_{fake}] \end{array}\right) + \left( \begin{array}{cc} 1 & 0\\ 0 & 1 \end{array}\right) \left( \begin{array}{cc} a_{E}[\{t_{1},v_{2}\}]\\ a_{E}[\{t_{fake},v_{1}\}] \end{array}\right) $$

and equation *m* = *m*_0_ + *Z*_*m*_Δ*m* is:

$$ \left( \begin{array}{cc} m[p_{1}]\\ m[p_{2}]\\ m[p_{3}] \end{array}\right) = \left( \begin{array}{cc} m_0[p_{1}]\\ m_0[p_{2}]\\ m_0[p_{3}] \end{array}\right) + \left( \begin{array}{cccc} -1 & 0 & -1 & 0\\ 0 & 1 & 0 & 0\\ 0 & 0 & 0& 1 \end{array}\right) \left( \begin{array}{cc} {\Delta}m[(p_{1},v_{1})]\\ {\Delta}m[(v_{1},p_{2})]\\ {\Delta}m[(p_{1},v_{2})]\\ {\Delta}m[(v_{2},p_{3})] \end{array}\right) $$

Let us assume that the marking of the net in Fig. 2a is *m*[*p*_1_]= 2, *m*[*p*_2_]= 0, *m*[*p*_3_]= 0, that one action was produced in *t*_1_, i.e. *σ*[*t*_1_]= 1, and it is available, i.e, *a*_*T*_[*t*_1_]= 1, and no event handler has fired. This corresponds to the state (*σ*[*t*_1_]= 1, *a*_*T*_[*t*_1_]= 1, *a*_*E*_[{*t*_1_,*v*_2_}]= 0, (Δ*m*[(*p*_1_,*v*_1_)]= 0,Δ*m*[(*v*_1_,*p*_2_)]= 0,Δ*m*[(*p*_1_,*v*_2_)]= 0, Δ*m*[(*v*_2_,*p*_3_)]= 0), (*m*[*p*_1_]= 2,*m*[*p*_2_]= 0,*m*[*p*_3_]= 0)). At this state, both event handlers, *v*_1_ and *v*_2_, are enabled and can fire. If *v*_2_ fires in an amount of 1, i.e. *x*= 1, then: *a*_*T*_[*t*_1_] and *m*[*p*_1_] are decreased by 1; *a*_*E*_[{*t*_1_,*v*_2_}] and Δ*m*[(*p*_1_,*v*_2_)] are increased by one; and Δ*m*[(*v*_2_,*p*_3_)] and *m*[*p*_3_] are increased by a nondeterministic quantity in the interval [1,2] (the value of *σ*[*t*_1_] remains unaltered as no actions are produced).

The graph in Fig. 2b shows the potential evolutions of the net under the assumption that event handlers fire in discrete amounts, i.e. all markings and actions are integers. The components of the vectors of states in the graph correspond to the variables (*m*[*p*_1_] *m*[*p*_2_] *m*[*p*_3_] *a*_*T*_[*t*_1_]). The arcs are labeled with the event handler that is fired. Remark that while the firing of *v*_1_ produces a deterministic change (one token consumed from *p*_1_ and one token produced in *p*_2_), the state change produced by the firing of *v*_2_ is nondeterministic (either one or two tokens can be produced in *p*_3_).

Notice that equations (7) account for the cumulative effect, and not the sequence, of the firings. In particular, the availability of tokens and actions consumed by the sequence of firings is not checked. This can lead to spurious solutions (Silva et al. 1998) in the state equations. Hence, equations (7) represent a necessary condition for the reachability of (*σ*, *a*_*T*_,*a*_*E*_,Δ*m*, *m*).

In order to account for linear relationships among the values of the initial marking, *m*_0_ is assumed to be a vector constrained as:

8 $$  J_{m}m_0\leq K_{m} $$

where *J*_*m*_ and *K*_*m*_ are real matrices of appropriate size. Note that the inequalities (8) can be used to account for the uncertain initial marking of a place, e.g. 10 ≤ *m*_0_[*p*_1_] ≤ 12, or to express linear constraints among markings, e.g. *m*_0_[*p*_1_] + *m*_0_[*p*_2_] = 5 and *m*_0_[*p*_4_] = 2*m*_0_[*p*_3_]. Equations (7) can be easily modified to take into account the relationships expressed by (8):

9 $$ \begin{array}{ll} SE_{{{\mathcal{N}}}_{V}}(\sigma,J_{m},K_{m}) = \{(&\sigma,a_{T},a_{E},{\Delta}m,m)| \\ & \sigma = a_{T} + Y_{\sigma} a_{E}\\ & A {\Delta}m \leq B a_{E}\\ & m = m_0 + Z_{m} {\Delta}m\\ & J_{m}m_0\leq K_{m}\} \end{array} $$

Although event handlers are not forced to fire, it is useful in some cases to consider only those states in which all the actions of given transitions have been executed. Let $T_{F}\subseteq T$ be the set of transitions whose actions must have been executed, i.e. the number of available actions of *t*_*j*_ ∈ *T*_*F*_ must be 0. In order to constrain (9) to such a set of states, the following equation can be added:

10 $$  a_{T}[t_{j}] = 0\ \ \forall\ t_{j}\in T_{F} $$

### Partial observability

In an event net, the marking change produced by the execution of an action is allowed to be nondeterministic. This is the case when there are several event handlers connected to a transition, or if the connected event handler has appropriate inequalities associated with it. The nondeterministic effect of the execution of actions can be used to develop nondeterministic models and, in particular, to model partially observable systems. The state equations (9) account for all the states that can be reached after the firing of event handlers. In the context of partial observability, these equations characterize the set of states that are consistent with a given observation of a partially observable system. The following example shows how a partially observable system can be modeled by an event net.

**Fig. 3:**
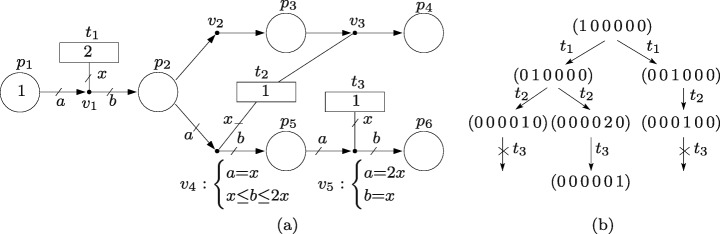
**a** Event net modeling a partially observable system. **b** Potential evolutions of the marking with discrete firings

For the sake of this example, let us assume that the event handlers of the net in Fig. 3a only fire in discrete amounts and that only the sequence of transitions whose actions are executed is observable. For clarity, the equations of event handlers that make all their labels equal are omitted, e.g. the equations of *v*_1_ are *a*=*b* and *a*=*x*, and they are therefore omitted (this same omission is made in the nets of the rest of the paper).

Thus, the observation of *t*_1_ corresponds to the firing of *v*_1_; the firing of *v*_2_ is unobservable (silent event) because it is not connected to a transition; the observation of *t*_2_ corresponds either to the firing of *v*_3_ or *v*_4_ because both handlers use the actions in *t*_2_ (in other words, *t*_2_ models events that cannot be distinguished by an observer); the observation of *t*_3_ corresponds to the firing of *v*_5_.

The graph in Fig. 3b shows the potential evolutions of the net with *m*_0_ = (1 0 0 0 0 0) and *σ* = (2 1 1), the components of the vectors in the graph correspond to the marking of places (*p*_1_*p*_2_*p*_3_*p*_4_*p*_5_*p*_6_). Initially, only *v*_1_ can fire and, hence, the execution of actions in *t*_1_ is the only event that can be observed. The observation of *t*_1_ means that a token was consumed from *p*_1_ and a token was produced in *p*_2_. Since *p*_2_ is the input place of *v*_2_ whose firing cannot be observed, a token in *p*_2_ can remain in *p*_2_ or *move silently* to *p*_3_. Thus, the set of markings consistent with the observation of *t*_1_ is {(0 1 0 0 0 0),(0 0 1 0 0 0)}, see second row of the graph. Assume that *t*_2_ is now observed, i.e. either *v*_3_ or *v*_4_ has fired. If the marking was (0 0 1 0 0 0), then *v*_3_ (the only enabled handler) has fired and that leads to marking (0 0 0 1 0 0). Otherwise, *v*_4_ has fired and that leads either to (0 0 0 0 1 0) or (0 0 0 0 2 0) because one firing of *v*_4_ can produce one or two tokens in *p*_5_. Thus, the set of markings consistent with the observation of the sequence of events *t*_1_ and *t*_2_ is {(0 0 0 0 1 0),(0 0 0 0 2 0),(0 0 0 1 0 0)}, see third row of the graph. Assume that *t*_3_ is now observed, i.e. *v*_5_ has fired. Notice that the firing of *v*_5_ requires two tokens in *p*_5_, thus, after the observation of *t*_1_ and *t*_2_, (0 0 0 0 2 0) is the only consistent marking at which *v*_5_ can fire. Consequently, the only sequence of markings consistent with the observation of *t*_1_, *t*_2_ and *t*_3_ is (1 0 0 0 0 0), (0 1 0 0 0 0), (0 0 0 0 2 0) and (0 0 0 0 0 1).

Notice that the state equations (7) can be used straightforwardly to compute the set of consistent markings with a given observation. For instance, for the observation of *t*_1_, *t*_2_ and *t*_3_ discussed above, the marking *m* = (0 0 0 0 0 1) is the only solution of:

$$ \sigma = a_{T} + Y_{\sigma} a_{E};\ A {\Delta}m \leq B a_{E};\ m = m_0 + Z_{m} {\Delta}m;\ \sigma=(2\ 1\ 1);\ a_{T}=(1\ 0\ 0) $$

where *σ* = (2 1 1) and *a*_*T*_ = (1 0 0) are the number of actions available at the beginning and at the end respectively. That is, each transition was observed once, i.e. *σ* − *a*_*T*_ = (1 1 1).

## Intensity nets

### Definition and state equations

This section introduces intensity nets, which can be denoted as *PST* nets, i.e. tokens in places *P* produce and consume intensities in transitions *T* through intensity handlers *S*. The intensity of a transition *t*_*j*_ is the speed at which actions are produced in *t*_*j*_. In other words, the number of actions produced at *t* is given by the integral over time of the intensity of *t*_*j*_. Intensity nets and event nets operate in a similar fashion. In fact, the changes in the intensities are produced by tokens in the intensity net in the same way that changes in the marking are produced by actions in the event net.

#### **Definition 5** (Intensity net)

An intensity net is a tuple ${{\mathcal {N}}}_{S}=(P,T,S,E_{S},C,D)$ where (*P*, *T*, *S*, *E*_*S*_) is a tripartite graph determining the net structure and (*C*, *D*) are matrices determining the potential intensity changes produced by the marking.

The set of vertices of the net is partitioned into three sets, *P* is the set of places, *T* is the set of transitions, and:

- *S* = {*s*_1_,…,*s*_*l*_,…} is a set of |*S*| intensity handlers.

Places and transitions model the same system features as in the event net. The intensity handlers are depicted as dots and model the different ways in which the tokens can generate intensities in the transitions.

The vertices of the net are connected by the edges in *E*_*S*_. Each pair of vertices can be connected by at most one edge. The set *E*_*S*_ is partitioned into two sets ${E_{S}^{T}}$ and ${E_{S}^{P}}$, where ${E_{S}^{T}}$ is a set of directed edges (or simply arcs) connecting transitions to intensity handlers and vice versa, and ${E_{S}^{P}}$ is a set of undirected edges (or simply edges) connecting places and intensity handlers. Thus, although both event handlers and intensity handlers are represented as dots, they can be easily distinguished by the arcs and edges that connect them to transitions and places. More formally:

- Every $e\in {E_{S}^{T}}$ is either an arc *e* = (*t*_*j*_,*s*_*l*_) from a transition *t*_*j*_ to a handler *s*_*l*_, or an arc *e* = (*s*_*l*_,*t*_*j*_) from a handler *s*_*l*_ to a transition *t*_*j*_.
- Every $e\in {E_{S}^{P}}$ is an edge *e* = {*p*_*i*_,*s*_*l*_} connecting a place *p*_*i*_ and a handler *s*_*l*_.

As in the event net, connections among places and transitions are not allowed. The following notation is used:

- ^*t*^*s*_*l*_ denotes the input transitions of *s*_*l*_, i.e. $^{t}s_{l}=\{t_{j}|(t_{j},s_{l})\in {E_{S}^{T}}\}$
- ${s_{l}^{t}}$ denotes the output transitions of *s*_*l*_, i.e. ${s_{l}^{t}}=\{t_{j}|(s_{l},t_{j})\in {E_{S}^{T}}\}$
- ^*s*^*t*_*j*_ denotes the input handlers of *t*_*j*_, i.e. $^{s}t_{j}=\{s_{l}|(s_{l},t_{j})\in {E_{S}^{T}}\}$
- ${t_{j}^{s}}$ denotes the output handlers of *t*_*j*_, i.e. ${t_{j}^{s}}=\{s_{l}|(t_{j},s_{l})\in {E_{S}^{T}}\}$
- ^*p*^*s*_*l*_ denotes the places connected to *s*_*l*_, i.e. $^{p}s_{l}=\{p_{i}|\{p_{i},s_{l}\}\in {E_{S}^{P}}\}$
- ${p_{i}^{s}}$ denotes the handlers connected to *p*_*i*_, i.e. ${p_{i}^{s}}=\{s_{l}|\{p_{i},s_{l}\}\in {E_{S}^{P}}\}$

As in the event net, the places in the intensity net contain tokens. These tokens can be used by the intensity handlers to produce intensities. A token is *active* if it is being used by an intensity handler, otherwise it is *idle*. While idle tokens are associated with places, active tokens are associated with edges. An intensity handler determines how much intensity is produced in its arcs as a function of the number of active tokens in its edges.

The state of the net is given by the variables associated with the net elements. Formally:

#### **Definition 6** (State)

The state of an intensity net ${{\mathcal {N}}}_{S}$ is given by the tuple (*m*, *μ*_*P*_,*μ*_*E*_,Δ*λ*, *λ*), where:

- $m\in \mathbb {R}^{|P|}_{\geq 0}$ is the marking, i.e. a vector indexed by *P* where *m*[*p*_*i*_] is the number of tokens in *p*_*i*_,
- $\mu _{P}\in \mathbb {R}^{|P|}_{\geq 0}$ is a vector indexed by *P* where *μ*_*P*_[*p*_*i*_] is the number of idle tokens in *p*_*i*_,
- $\mu _{E}\in \mathbb {R}^{|{E_{S}^{P}}|}_{\geq 0}$ is a vector indexed by ${E_{S}^{P}}$ where *μ*_*E*_[{*p*_*i*_,*s*_*l*_}] is the number of active tokens of *p*_*i*_ being used by *s*_*l*_,
- ${\Delta }\lambda \in \mathbb {R}^{|{E_{S}^{T}}|}_{\geq 0}$ is a vector indexed by ${E_{S}^{T}}$ where Δ*λ*[(*t*_*j*_,*s*_*l*_)] is a decrease of intensity in *t*_*j*_ produced by *s*_*l*_, and Δ*λ*[(*s*_*l*_,*t*_*j*_)] is an increase of intensity in *t*_*j*_ produced by *s*_*l*_,
- $\lambda \in \mathbb {R}^{|T|}_{\geq 0}$ is a vector indexed by *T* where *λ*[*t*_*j*_] is the intensity in *t*_*j*_.

The number of tokens in a place *p*_*i*_ is equal to the number of its idle tokens plus the number of its active tokens:

11 $$  m[p_{i}]=\mu_{P}[p_{i}]+\sum\limits_{s_{l} \in {p_{i}^{s}}} \mu_{E}[\{p_{i},s_{l}\}]\ \ \forall\ p_{i}\in P $$

An intensity handler is said to be working when it is producing intensities. When an intensity handler *s*_*l*_ ∈ *S* starts working, the number of idle tokens in ^*p*^*s*_*l*_ decreases, the number of active tokens in its edges increases (such tokens start being used by the handler), and intensities are produced in its arcs. Conversely, when an intensity handler *s*_*l*_ stops working, the number of idle tokens in ^*p*^*s*_*l*_ increases (i.e. they are released by the handler), the number of active tokens becomes 0, and no intensities are produced in its arcs. Thus (in contrast to the firing of event handlers whose firing cannot be reversed once it has occurred) intensity handlers are allowed to start and stop working (or to increase and decrease their working rates), thus allocating tokens as active tokens and releasing them as idle tokens over time.

The relation between the number of active tokens and the intensities produced are given by a set of inequalities associated with each intensity handler *s*_*l*_ ∈ *S*. The coefficients of these inequalities can be captured by two matrices (*C*_*l*_,*D*_*l*_) of real numbers and same number of rows. The columns of *C*_*l*_ are indexed by the arcs connecting *s*_*l*_ to transitions. The columns of *D*_*l*_ are indexed by the edges connecting places to *s*_*l*_. Matrix *C*(*D*) is obtained by arranging all the matrices *C*_*l*_(*D*_*l*_) *diagonally*.

Each transition *t*_*j*_ is assigned a default (or nominal) intensity *λ*_0_[*t*_*j*_]. Thus, the intensity *λ*[*t*_*j*_] in a transition *t*_*j*_ is equal to *λ*_0_[*t*_*j*_] plus the positive changes in intensity minus the negative changes in intensity:

12 $$  \lambda[t_{j}]=\lambda_{0}[t_{j}] {-} \sum\limits_{s_{l}\in {t_{j}^{s}}} {\Delta}\lambda[(t_{j},s_{l})] {+}\sum\limits_{s_{l}\in ^{s}t_{j}} {\Delta}\lambda[(s_{l},t_{j})]\ \ \forall\ t_{j}\in T $$

The number of active tokens, $\mu _{w}\in \mathbb {R}_{\geq 0}^{|^{p}s_{l}|}$ indexed by the edges of *s*_*l*_, being used by an intensity handler *s*_*l*_, and the intensities, ${\Delta }\lambda _{w}\in \mathbb {R}_{\geq 0}^{|^{t}s_{l}|+|{s_{l}^{t}}|}$ indexed by the arcs of *s*_*l*_, produced by *s*_*l*_ are related by the matrices *C*_*l*_ and *D*_*l*_ as follows:

13 $$  C_{l} {\Delta}\lambda_{w} \leq D_{l} \mu_{w} $$

If **1***μ*_*w*_ + **1**Δ*λ*_*w*_ > 0, then *s*_*l*_ is said to be working. Similarly to event handlers, intensity handlers are not forced to work. When a number of intensity handlers work simultaneously, they share the tokens in places and collaborate in the production of intensities. In a similar way to (4), the inequalities (13) allow the modeling of uncertainty in the intensity changes produced by the active tokens.

Similarly to (7), the state equations of the intensity net determine the potential states of the net for a given marking *m* and default intensities *λ*_0_:

#### **Proposition 2** (State equations)

Let the state of an intensity net ${{\mathcal {N}}}_{S}$ be (*m*, *m*,0,0,*λ*_0_), i.e. *m* idle tokens are available and no intensity handler is working. Every state (*m*, *μ*_*P*_,*μ*_*E*_,Δ*λ*, *λ*) reachable from (*m*, *m*,0,0,*λ*_0_) belongs to $SE_{{{\mathcal {N}}}_{S}}(m,\lambda _{0})$ where:

14 $$ \begin{array}{ll} SE_{{{\mathcal{N}}}_{S}}(m,\lambda_{0}) = \{(&m,\mu_{P},\mu_{E},{\Delta}\lambda,\lambda)| \\ & m = \mu_{P} + Y_{m} \mu_{E}\\ & C {\Delta}\lambda \leq D \mu_{E}\\ & \lambda = \lambda_{0} + Z_{\lambda} {\Delta}\lambda\} \end{array} $$

where *Y*_*m*_ and *Z*_*λ*_ are matrices determined by the net structure:

- *Y*_*m*_ is a matrix with rows indexed by *P*, columns indexed by ${E_{S}^{P}}$, and such that $Y_{m}[p_{i},\{p_{i},s_{l}\}]=1\ \forall \{p_{i},s_{l}\}\in {E_{S}^{P}}$ and the rest of the elements in *Y*_*m*_ are 0,
- *Z*_*λ*_ is a matrix with rows indexed by *T*, columns indexed by ${E_{S}^{T}}$, and such that $Z_{\lambda }[t_{j},(t_{j},s_{l})]=-1\ \forall (t_{j},s_{l}) \in {E_{S}^{T}}$, $Z_{\lambda }[t_{j},(s_{l},t_{j})]=1\ \forall (s_{l},t_{j}) \in {E_{S}^{T}}$ and the rest of the elements in *Z*_*λ*_ are 0,

and *μ*_*P*_, *μ*_*E*_, Δ*λ* and *λ* are nonnegative variables.

Similarly to (7), the equations *m*=*μ*_*P*_ + *Y*_*m*_*μ*_*E*_, *C*Δ*λ*≤*Dμ*_*E*_, *λ*=*λ*_0_ + *Z*_*λ*_Δ*λ* in (14) are the matrix forms of (11), (13) and (12) respectively, and thus, must hold at every state (*m*, *μ*_*P*_,*μ*_*E*_,Δ*λ*, *λ*) reachable from (*m*, *m*,0,0,*λ*_0_). As in (7), equations (14) account for the cumulative intensities produced by the handlers, and hence, $SE_{{{\mathcal {N}}}_{S}}(m,\lambda _{0})$ can contain spurious solutions.

As in (8), inequalities can be considered to model linear relationships among the values of the default intensities, *λ*_0_. Let us assume that *λ*_0_ is a vector constrained as:

15 $$  J_{\lambda}\lambda_{0}\leq K_{\lambda} $$

where *J*_*λ*_ and *K*_*λ*_ are real matrices of appropriate size. Similarly to (8), the inequalities (15) can be used to model the uncertain default intensity of a transition, or to establish linear constraints among default intensities of transitions. Equations (14) can be easily modified to take into account the relationships expressed by (15):

16 $$ \begin{array}{ll} SE_{{{\mathcal{N}}}_{S}}(m,J_{\lambda},K_{\lambda}) = \{(&m,\mu_{P},\mu_{E},{\Delta}\lambda,\lambda)| \\ & m = \mu_{P} + Y_{m} \mu_{E}\\ & C {\Delta}\lambda \leq D \mu_{E}\\ & \lambda = \lambda_{0} + Z_{\lambda} {\Delta}\lambda\\ & J_{\lambda}\lambda_{0}\leq K_{\lambda}\} \end{array} $$

Although intensity handlers are not forced to work, it is useful in some cases to consider only those states in which all the tokens of given places are active. Let $P_{F}\subseteq P$ be the set of places whose tokens must be active, i.e. the number of idle tokens of *p*_*i*_ ∈ *P*_*F*_ must be 0. In order to constrain (16) to such a set of states, the following equation can be added:

17 $$  \mu_{P}[p_{i}] = 0\ \ \forall\ p_{i}\in P_{F} $$

### Modeling capabilities

**Fig. 4:**
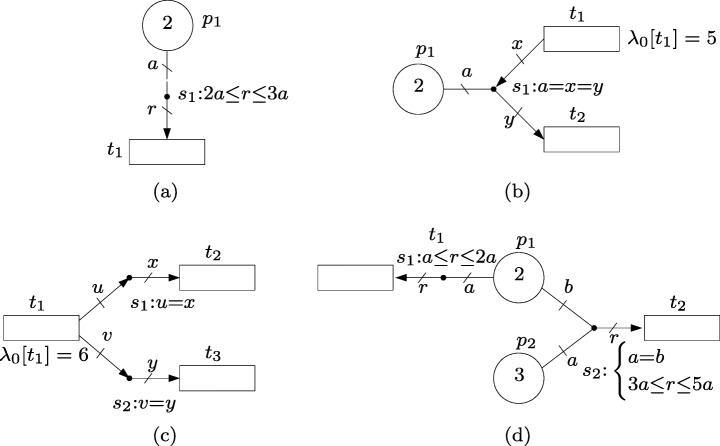
Modeling capabilities of intensity nets

Figure 4 shows some of the modeling capabilities of intensity nets. The default intensity, *λ*_0_[*t*], of a transition, *t*, can be written next to the transition, see Fig. 4b (default intensities equal to 0 are omitted in the figures). As in the event nets, labels are associated with arcs and edges to represent amounts of produced/consumed intensities and number of active tokens.

The intensity net in Fig. 4a has one place *p*_1_, one transition *t*_1_ and one intensity handler *s*_1_. The inequality associated with *s*_1_ establishes that the intensity Δ*λ*[(*s*_1_,*t*_1_)] produced in the arc (*s*_1_,*t*_1_) by *s*_1_ must satisfy 2*μ*_*E*_[{*p*_1_,*s*_1_}]≤Δ*λ*[(*s*_1_,*t*_1_)]≤ 3*μ*_*E*_[{*p*_1_,*s*_1_}] where *μ*_*E*_[{*p*_1_,*s*_1_}] is the number of active tokens in {*p*_1_,*s*_1_} (notice that given that *m*[*p*_1_]= 2, the number of active tokens is upper bounded by 2). The actual value of Δ*λ*[(*s*_1_,*t*_1_)] is selected nondeterministically in this interval. Since the default intensity of *t*_1_ is 0 and (*s*_1_,*t*_1_) is the only arc connected to *t*_1_, it holds *λ*[*t*_1_]=Δ*λ*[(*s*_1_,*t*_1_)] what establishes the rate at which actions will be produced in *t*_1_.

The intensity handler *s*_1_ in Fig. 4b makes use of the active tokens in *p*_1_ to decrease the intensity in *t*_1_ and increase the intensity in *t*_2_. This can be seen as an *intensity transfer* from one transition to the other. According to the equations associated with *s*_1_, which can be rewritten as *μ*_*E*_[{*p*_1_,*s*_1_}] = Δ*λ*[(*t*_1_,*s*_1_)] = Δ*λ*[(*s*_1_,*t*_2_)], the amount of this transfer is equal to the number of active tokens, *μ*_*E*_[{*p*_1_,*s*_1_}], which in this case is at most 2 given that *m*[*p*_1_] = 2. Thus, if there is one active token, i.e. *μ*_*E*_[{*p*_1_,*s*_1_}] = 1, according to the state equations (14) the resulting intensities will be *λ*[*t*_1_] = *λ*_0_[*t*_1_] −Δ*λ*[(*t*_1_,*s*_1_)] = *λ*_0_[*t*_1_] − *μ*_*E*_[{*p*_1_,*s*_1_}] = 5 − 1 = 4 and *λ*[*t*_2_] = *λ*_0_[*t*_2_] + Δ*λ*[(*s*_1_,*t*_2_)] = *λ*_0_[*t*_2_] + *μ*_*E*_[{*p*_1_,*s*_1_}] = 0 + 1 = 1.

The net in Fig. 4c shows how the intensity of one transition, *t*_1_, can be used to produce intensity in other transitions, *t*_2_ and *t*_3_. For this net, the state equations (14) become *λ*[*t*_1_] = *λ*_0_[*t*_1_]−Δ*λ*[(*t*_1_,*s*_1_)]−Δ*λ*[(*t*_1_,*s*_2_)], *λ*[*t*_2_] = *λ*_0_[*t*_2_]+Δ*λ*[(*s*_1_,*t*_2_)], *λ*[*t*_3_] = *λ*_0_[*t*_3_]+Δ*λ*[(*s*_2_,*t*_3_)], Δ*λ*[(*t*_1_,*s*_1_)] = Δ*λ*[(*s*_1_,*t*_2_)], Δ*λ*[(*t*_1_,*s*_2_)] = Δ*λ*[(*s*_2_,*t*_3_)]. Given that *λ*_0_[*t*_1_] = 6 and *λ*_0_[*t*_2_] = *λ*_0_[*t*_3_] = 0, these state equations reduce to *λ*[*t*_1_]+*λ*[*t*_2_]+*λ*[*t*_3_] = 6 what summarizes the potential intensities of the net.

The net in Fig. 4d models a choice in place *p*_1_, i.e. each token in *p*_1_ can be used either to produce an intensity within the interval [1,2] in *t*_1_, or synchronize with a token in *p*_2_ to produce an intensity within the interval [3,5] in *t*_2_.

## Flexible nets

This section introduces Flexible Nets (FNs), which can be denoted as *PHT* nets, i.e. places *P* and transitions *T* are connected by event and intensity handlers. Roughly, an FN consists of an event net and an intensity net that have the same set of places and the same set of transitions.

### **Definition 7** (Flexible net)

A Flexible Net (FN) is a tuple $\mathcal {N}$ = (*P*, *T*, *V*, *E*_*V*_,*A*, *B*, *S*, *E*_*S*_,*C*, *D*) where (*P*, *T*, *V*, *E*_*V*_,*A*, *B*) is an event net and (*P*, *T*, *S*, *E*_*S*_,*C*, *D*) is an intensity net.

In an FN, the event net determines the way actions produce marking changes, and the intensity net determines the way tokens produce intensity changes. The inequalities associated with handlers allow the modeler to cover a range of relationships between “actions and tokens” and “tokens and intensities”. Thus, handlers can be seen as a flexible layer between places and transitions that offers the possibility to model uncertainties in both the way actions produce marking changes, and the way tokens produce intensity changes.

**Fig. 5:**
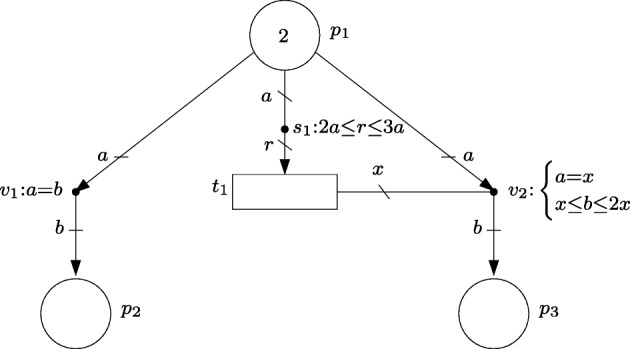
FN resulting of combining the event net in Fig. 2a and the intensity net in Fig. 4a

The FN in Fig. 5 is composed of the event net in Fig. 2a and the intensity net in Fig. 4a. While the event net determines the marking changes produced by the firing of event handlers, the intensity net establishes the rate at which actions are created in *t*_1_. Notice that the firing of *v*_2_ implies the execution of actions in *t*_1_, i.e. actions need to be produced in *t*_1_ so that *v*_2_ can fire. On the other hand, *v*_1_ is not connected to any transitions and, thus, it can fire when there is a positive marking in *p*_1_. It should be noted that this is not equivalent to an immediate transition in Petri nets, since the firing of *t*_2_ is not forced to happen as soon as the marking of *p*_1_ is positive, its firing can occur at any time at which the marking of *p*_1_ is positive.

In order to compute the number of actions produced in transitions, the number of actions produced in the intensity arcs will be computed first. Let Δ*σ*(*τ*) denote the number of actions produced in the intensity arcs until time *τ* (Δ*σ*[*e*](*τ*) with $e\in {E_{S}^{T}}$ denotes the number of actions produced in *e*). The value of Δ*σ*(*τ*) is defined as the integral of Δ*λ* over time:

18 $$  {\Delta}\sigma(\tau)={\int}_{0}^{\tau} \! {\Delta}\lambda(s) \mathrm{d}s $$

The overall number of actions, *σ*[*t*_*j*_](*τ*), produced in a transition *t*_*j*_ can be computed by integrating *λ*[*t*_*j*_], or equivalently, by making use of *Z*_*λ*_, see (14), and Δ*σ*(*τ*):

19 $$  \sigma(\tau) = \lambda_{0}\tau + Z_{\lambda} {\Delta}\sigma(\tau)\\ $$

In addition to the state variables of the event and intensity net, Δ*σ* is included in the tuple of variables defining the state of the FN.

### **Definition 8** (State)

The state **x** of an FN is given by the tuple **x** = (*m*, *μ*_*P*_,*μ*_*E*_,Δ*λ*, *λ*, Δ*σ*, *σ*, *a*_*T*_,*a*_*E*_,Δ*m*).

All the state variables are time dependent. For the sake of clarity, the time dependency will be omitted when it is clear from the context, e.g. *m*(*τ*) is shortened to *m*. At time 0 it holds Δ*σ* = 0, *σ* = 0, *a*_*T*_ = 0, *a*_*E*_ = 0, Δ*m* = 0, i.e. the initial state can be written as: (*m*, *μ*_*P*_,*μ*_*E*_,Δ*λ*, *λ*,0,0,0,0,0).

By making use of $SE_{{{\mathcal {N}}}_{V}}(\sigma ,J_{m},K_{m})$ in (9), $SE_{{{\mathcal {N}}}_{S}}(m,J_{\lambda },K_{\lambda })$ in (16), (18) and (19), it is possible to write a set of equations that any potential state at time *τ* must satisfy.

### **Proposition 3** (State equations)

Let ${{\mathcal {N}}}$ be an FN with initial marking *m*_0_ satisfying *J*_*m*_*m*_0_ ≤ *K*_*m*_, and default intensities *λ*_0_ satisfying *J*_*λ*_*λ*_0_ ≤ *K*_*λ*_. Every state (*m*, *μ*_*P*_, *μ*_*E*_,Δ*λ*, *λ*,Δ*σ*, *σ*, *a*_*T*_,*a*_*E*_,Δ*m*) reachable at time *τ* belongs to $SE_{{{\mathcal {N}}}}(\tau ,J_{m},K_{m},J_{\lambda },K_{\lambda })$ where:

20 $$ \begin{array}{ll} SE_{{{\mathcal{N}}}}&(\tau,J_{m},K_{m},J_{\lambda},K_{\lambda}) = \{(m,\mu_{P},\mu_{E},{\Delta}\lambda,\lambda,{\Delta}\sigma,\sigma,a_{T},a_{E},{\Delta}m)| \\ & m = \mu_{P} + Y_{m} \mu_{E};\ C {\Delta}\lambda \leq D \mu_{E};\ \lambda = \lambda_{0} + Z_{\lambda} {\Delta}\lambda; J_{\lambda}\lambda_{0}\leq K_{\lambda}\\ & {\Delta}\sigma=\displaystyle{\int}_{0}^{\tau} \! {\Delta}\lambda(s) \mathrm{d}s;\ \sigma = \lambda_{0}\tau + Z_{\lambda} {\Delta}\sigma\\ & \sigma = a_{T} + Y_{\sigma} a_{E};\ A {\Delta}m \leq B a_{E};\ m = m_0 + Z_{m} {\Delta}m;\ J_{m}m_0\leq K_{m} \} \end{array} $$

where every variable is nonnegative.

This way, an FN is a continuous time model where time, denoted as *τ*, is the independent variable and all the state variables are nonnegative reals.

Equations (20) can be interpreted as follows: at a given time *τ*, some of the produced actions (*σ*) are available (*a*_*T*_), and the rest (*a*_*E*_) were executed before *τ*. The executed actions produced marking changes (Δ*m*) which resulted in the marking *m* in places at *τ*. Some of the tokens in *m* are active (*μ*_*E*_) and the rest are idle (*μ*_*P*_). Active tokens produce intensity changes (Δ*λ*) which result in overall intensities (*λ*) in transitions at *τ*. The integral of the intensity changes and overall intensities over time after *τ* will produce more actions (*σ*), i.e. *σ* is produced as time elapses. This behavior repeats over time: when a new marking is reached, intensities are updated, which can lead to the production and execution of new actions, which consequently results in a new marking.

As in the event and intensity nets, constraints (10) and (17) can be added to (20) to force the execution of actions and the activity of places.

## Exploiting uncertainty

The state equations (20) account for all the potential states of the net at time *τ*. In order to facilitate the analysis of FNs, a set of necessary reachability conditions was developed (Júlvez et al. 2018). These conditions consist of linear and quadratic inequalities that all the solutions of (20) must satisfy during the interval [0,*τ*]. In order to obtain a time trajectory of the state, i.e. values of the state at different time instants *τ*_1_, *τ*_2_, *τ*_3_, …, two methods are considered:

1. The first method consists of developing a unique set of necessary reachability conditions that combines the reachability conditions of each interval [0,*τ*_1_], [*τ*_1_,*τ*_2_],[*τ*_2_,*τ*_3_],…, in such a way that all the states that satisfy the constraints at the end of a given interval are taken as potential initial states for the next interval (see Júlvez et al. 2018 for details). Once this set of constraints is obtained, a particular trajectory of the FN can be computed by adding an objective function to such a set of constraints, and by solving the resulting programming problem.
2. The second method follows a model predictive control (MPC) (Kouvaritakis and Cannon 2016) approach. According to this approach, the programming problem described in the first method is solved over the intervals [0,*τ*_1_],[*τ*_1_,*τ*_2_],…,[*τ*_*n*− 1_,*τ*_*n*_] where *τ*_*n*_ is the prediction horizon. Then, the state obtained at *τ*_1_ is taken as the initial state and a programming problem over the intervals [*τ*_1_,*τ*_2_],[*τ*_2_,*τ*_3_],…,[*τ*_*n*_,*τ*_*n*+ 1_] is defined and solved. This procedure can be repeated with the subsequent intervals.

It should be noted that solving convex quadratic programming problems is required by both methods above. Given that the computational complexity required to solve such problems is polynomial, the proposed computational methods can be applied to large FNs. The following subsections present some of the modeling, analysis and control capabilities of FNs by modeling a linear system with uncertain parameters, a resource allocation system and a system with control actions.

### Linear system with uncertain parameters

**Fig. 6:**
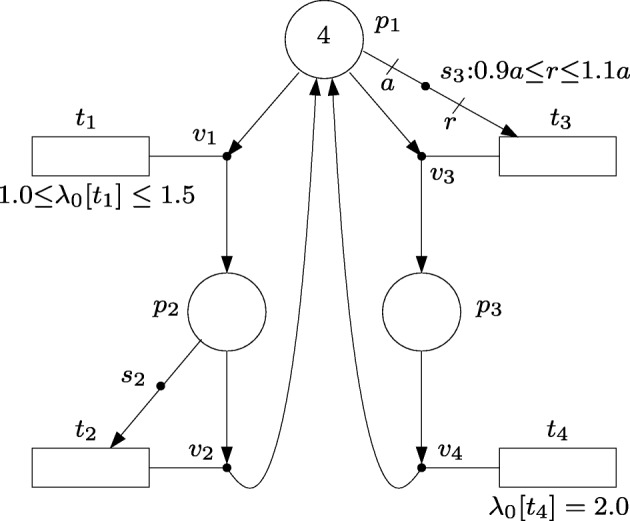
FN with uncertain dynamics modeled by the default intensity of *t*_1_ and the inequalities of *s*_3_

The FN in Fig. 6 models a linear system with uncertain dynamics. More precisely, if we assume that all the tokens are forced to be active and all the actions to be executed, the rate at which the marking changes can be expressed as:

21 $$ \begin{array}{@{}rcl@{}} \dot{m}[p_{1}] & =& -q - hm[p_{1}] + m[p_{2}] + 2 \end{array} $$

22 $$ \begin{array}{@{}rcl@{}} \dot{m}[p_{2}] & =& q - m[p_{2}] \end{array} $$

23 $$ \begin{array}{@{}rcl@{}} \dot{m}[p_{3}] & =& hm[p_{1}] - 2 \end{array} $$

where *q* and *h* are uncertain parameters but known to be in the intervals: *q* ∈ [1.0,1.5], *h* ∈ [0.9,1.1]. Thus, any potential time trajectory of the system will satisfy (21) with values of *q* and *h* within the given intervals. The uncertain parameter *q* is modeled by the default intensity of *t*_1_, and *h* is modeled by the inequalities of *s*_3_. Notice that the FN in Fig. 6 combines transitions with constant speed, e.g. *t*_4_, transitions whose speed is proportional to the marking of a place, e.g. *t*_2_, and uncertain parameters.

**Fig. 7:**
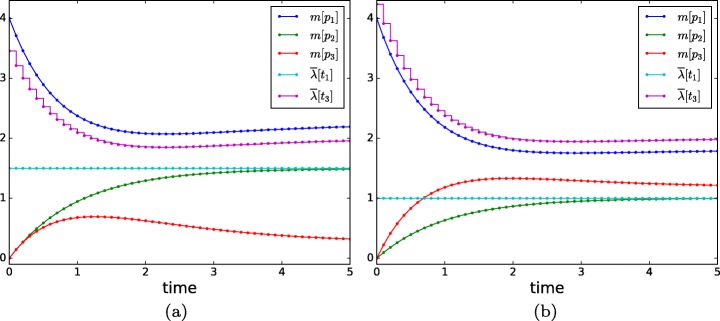
Time evolution of the FN in Fig. 6 when *m*[*p*_3_] is minimized (**a**) and maximized (**b**)

Let the initial marking be *m*_0_[*p*_1_]= 4, *m*_0_[*p*_2_]= 0, and *m*_0_[*p*_3_]= 0. Figure 7 shows the time trajectories of the marking and the intensities of *t*_1_ and *t*_3_ under different objective functions (notice that the intensity of *t*_2_ is equal to *m*[*p*_2_], and the intensity of *t*_4_ is constant and equal to 2). The trajectories have been obtained by an MPC approach with a sample time (or interval) of 0.1 time units and a prediction horizon of one sample time. This means that initially, i.e. at time 0.0, the programming problem (Júlvez et al. 2018) is defined and solved over the time interval [0.0, 0.1]. The solution of the problem is taken as the state of the system at time 0.1. Then, the programming problem is defined and solved over the interval [0.1, 0.2], and the procedure is repeated.

The trajectory in Fig. 7a is obtained by the objective function “*minm*[*p*_3_]”, i.e. the goal is to minimize the marking of *p*_3_ at the end of each interval. In the plots, $\bar {\lambda }[t_{j}]$ denotes the average intensity of *t*_*j*_ during each interval. For such an objective, the solution of the programming problem sets the uncertain parameters to *λ*_0_[*t*_1_]= 1.5 and 0.9*a*=*r* (which results in *λ*[*t*_3_]= 0.9*m*[*p*_1_]). This setting minimizes the flow directed from *p*_1_ to the branch composed of *v*_3_ and *v*_4_. As expected, the intensity of *t*_1_ and *t*_2_ (*t*_3_ and *t*_4_) is the same at steady state.

The trajectory in Fig. 7b is obtained by the objective function “*maxm*[*p*_3_]”. For such an objective, the solution of the programming problem sets *λ*_0_[*t*_1_]= 1.0 and *r*= 1.1*a* (which results in *λ*[*t*_3_] = 1.1*m*[*p*_1_]). This setting maximizes the flow directed from *p*_1_ to the branch composed of *v*_3_ and *v*_4_.

### Resource allocation

**Fig. 8:**
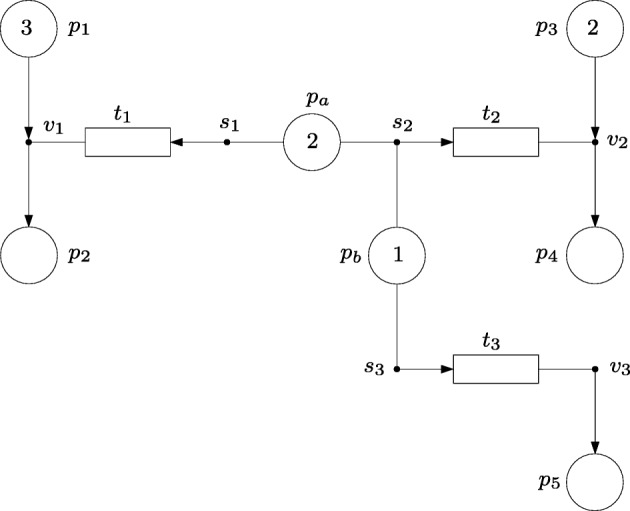
FN modeling a resource allocation system with three production lines and two shared resources

The FNs in Fig. 8 models a dynamic system in which shared resources can be allocated to different production lines. Such a net shows how the tokens of a given place can activate different processes (those places have several intensity edges) and can cooperate with active tokens of other places. Namely, there are two types of resources, *p*_*a*_ and *p*_*b*_, and three production lines, *t*_1_, *t*_2_ and *t*_3_. The production line associated with *t*_1_(*t*_2_) uses the raw material modeled by the tokens in *p*_1_(*p*_3_) and produces items modeled by the tokens in *p*_2_(*p*_4_). The production line associated with *t*_3_ produces tokens in *p*_5_ and it is assumed that it requires no raw material (or equivalently, this raw material is inexhaustible). In order to operate, the production line associated with *t*_1_(*t*_3_) requires the allocation of resources of type *p*_*a*_(*p*_*b*_). The speed of these production lines, *t*_1_ and *t*_3_, is proportional to the number of tokens allocated to them. The operation of production line *t*_2_ requires the cooperation of both resources, *p*_*a*_ and *p*_*b*_, i.e. tokens of both resources must synchronize in equal amounts to make *t*_2_ work. The speed of *t*_2_ is equal to the number of tokens of *p*_*a*_ (or *p*_*b*_) allocated to this production line.

While the event handlers, *v*_1_, *v*_2_ and *v*_3_ determine the relationship between the input and output material of the production lines, the intensity handlers specify the speed of these lines according to the number of active tokens assigned to each line. In particular, the intensity edges {*p*_*a*_,*s*_1_} and {*p*_*a*_,*s*_2_} model the fact that the active tokens of *p*_*a*_ can be used either by *s*_1_ or *s*_2_. In a similar way, the fact that the active tokens of *p*_*b*_ can be used either by *s*_2_ or *s*_3_ is modeled by the intensity edges {*p*_*b*_,*s*_2_} and {*p*_*b*_,*s*_3_}. This way, *s*_2_ is the intensity handler responsible for the synchronization of resources for *t*_2_. The actions of all transitions are forced to be executed in order to model the active tokens to make the production lines work. Note that the graphical representation of the system by an FN is reasonably clear and compact. If the system were modeled by a classical Petri net, each transition would have to be split into several transitions that would each model the acquisition and release of the resources and the speed of the production lines. This would lead to a more complex graphical notation and, potentially, to more involved analysis methods.

Let the initial marking of the net be *m*_0_[*p*_1_]= 3, *m*_0_[*p*_2_]= 0, *m*_0_[*p*_3_]= 2, *m*_0_[*p*_4_]= 0, *m*_0_[*p*_5_]= 0, *m*_0_[*p*_*a*_]= 2 and *m*_0_[*p*_*b*_]= 1, i.e. there are two copies of resource type *p*_*a*_ and one copy of resource type *p*_*b*_. Assume that the goal is to compute how the resources must be allocated over time so that the objective function $\bar {m}[p_{2}]{+}0.5\bar {m}[p_{4}]{+}0.25\bar {m}[p_{5}]$, where $\bar {m}[p_{i}]$ denotes the average marking of *p*_*i*_, is maximized. In words, this objective function implies that the goal is to maximize the production of all items giving priority to the products of type *p*_2_, then *p*_4_ and finally *p*_5_.

This resource allocation problem can be solved by a single programming problem (see first method in Section 5) that makes use of the reachability constraints in Júlvez et al. (2018) and the mentioned objective function. More precisely, in order to obtain time trajectories, 90 intervals, each of 0.05 time units, will be considered.

**Fig. 9:**
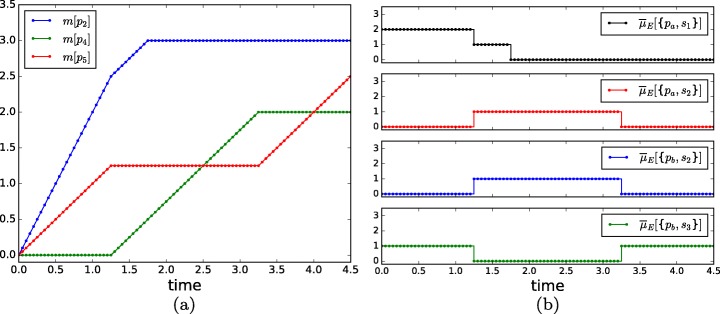
Time trajectories of the marking (**a**) of the net in Fig. 8, and of the number of resources allocated to the transitions (**b**)

The time trajectories of the marking and the allocated resources are shown in Fig. 9a and b respectively. Four time periods with different resource allocations (or operation modes) can be distinguished in these figures. The first period, from time 0 to 1.25, allocates the two tokens of *p*_*a*_ to *s*_1_. This gives a high yield in the production of the items in *p*_2_ which has the highest priority. Given that the two tokens of *p*_*a*_ are used by *s*_1_ during this first period, the token in *p*_*b*_ cannot be used by *s*_2_, and hence it is used by *s*_3_ to produce the items in *p*_5_, which has the lowest priority. During the second time period, from time 1.25 to 1.75, one token of *p*_*a*_ is used by *s*_1_, and the other token of *p*_*a*_ is synchronized by *s*_2_ with the token of *p*_*b*_ to operate *t*_2_ and produce the items in *p*_4_, which has medium priority. As a result, the speed of *t*_1_(*t*_2_)(*t*_3_) is 1(1)(0) during the second time period. At time 1.75, the marking of *p*_1_ becomes 0, and hence, the active token of *p*_*a*_ allocated to *s*_1_ is released and becomes idle. Thus, during the third period, from time 1.75 to 3.25, only *t*_2_ is working. At time 3.25, the marking of *p*_3_ becomes 0, and hence the two tokens of *p*_*a*_ become idle. During the fourth period, from time 3.25 onward, only the token of *p*_*b*_ is active, and is employed by *s*_3_ to operate *t*_3_.

### Control actions

Control actions can be modeled in FNs by means of default intensities. This section demonstrates the ability of FNs to model and solve a control problem in which the control action is dynamically constrained.

**Fig. 10:**
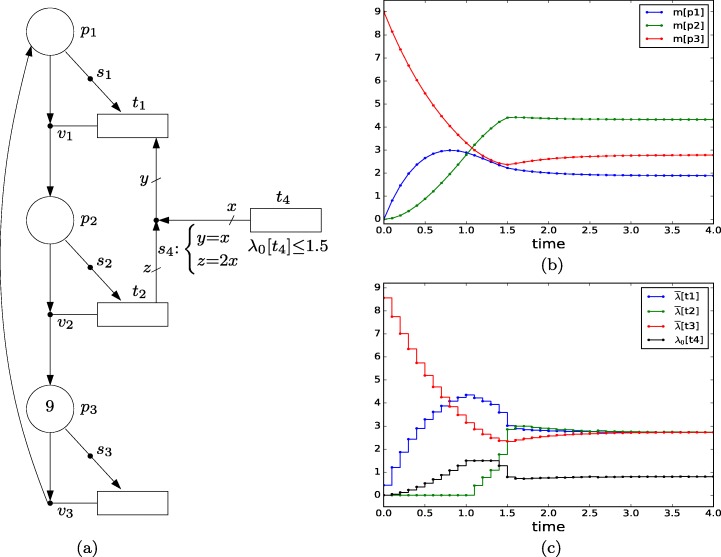
**a** FN with control action modeled by *λ*_0_[*t*_4_]. **b** Time evolution of the marking. **c** Time evolution of the average intensities during each interval, $\bar {\lambda }[t_{1}]$, $\bar {\lambda }[t_{2}]$, $\bar {\lambda }[t_{3}]$, and control action *λ*_0_[*t*_4_]

Figure 10a depicts a net with three places and four transitions. All the tokens are forced to be active, and all the actions are forced to be executed. The initial marking is *m*_0_[*p*_1_]=*m*_0_[*p*_2_]= 0 and *m*_0_[*p*_3_]= 9. The default intensities of *t*_1_, *t*_2_ and *t*_3_ are 0. The default intensity of *t*_4_, *λ*_0_[*t*_4_], models the only control action that can be applied to the system, and is constrained to the interval [0,1.5]. Given that the equations associated with *s*_4_ are *s*_4_:*y*=*x*;*z*= 2*x*, each intensity unit in *t*_4_ increases the intensity in *t*_1_, *λ*[*t*_1_], by one unit and decreases the intensity in *t*_2_, *λ*[*t*_2_], by two units. This way, the same control action is used for the intensities of *t*_1_ and *t*_2_. Thus, the intensities in transitions satisfy *λ*[*t*_1_]=*m*[*p*_1_] + *λ*_0_[*t*_4_], *λ*[*t*_2_]=*m*[*p*_2_] − 2*λ*_0_[*t*_4_], *λ*[*t*_3_]=*m*[*p*_3_]. Notice that the input action *λ*_0_[*t*_4_] is not only statically constrained by *λ*_0_[*t*_4_]≤ 1.5, but also dynamically constrained by *λ*_0_[*t*_4_]≤ 0.5*m*[*p*_2_] (if this constraint is violated then *λ*[*t*_2_] becomes negative).

The evolution of the system can be described by the following differential equations:

24 $$ \begin{array}{@{}rcl@{}} \dot{m}[p_{1}] & =& \lambda[t_{3}]-\lambda[t_{1}] = m[p_{3}] - m[p_{1}] - \lambda_{0}[t_{4}] \end{array} $$

25 $$ \begin{array}{@{}rcl@{}} \dot{m}[p_{2}] & =& \lambda[t_{1}]-\lambda[t_{2}] = m[p_{1}] - m[p_{2}] + 3\lambda_{0}[t_{4}] \end{array} $$

26 $$ \begin{array}{@{}rcl@{}} \dot{m}[p_{3}] & =& \lambda[t_{2}]-\lambda[t_{3}] = m[p_{2}] - 2\lambda_{0}[t_{4}] - m[p_{3}] \end{array} $$

where all the variables are nonnegative and *λ*_0_[*t*_4_]≤ 1.5.

Consider the objective function *min* (*m*[*p*_1_] − 1)^2^ + (*m*[*p*_2_] − 4)^2^. Notice that in this system the invariant *m*[*p*_1_] + *m*[*p*_2_] + *m*[*p*_3_] = 9 holds, then, the control objective is to drive the system to a marking that is as close as possible to the target marking (1,4,4). Figure 10b and c show the trajectories obtained by MPC with a sample time of 0.1 time units and a prediction horizon of one step. Initially, the value of *λ*_0_[*t*_4_] is low as it is constrained by *m*[*p*_2_], which initially is 0. Then, *λ*_0_[*t*_4_] increases so that *m*[*p*_2_] increases and *m*[*p*_1_] decreases. At time 1.0, *λ*_0_[*t*_4_] hits the constraint 1.5 where it is kept constant for 0.4 time units. Then, *λ*_0_[*t*_4_] decreases in order to approach further the target marking. At steady state, the average intensities of *t*_1_, *t*_2_ and *t*_3_ are the same and equal to 2.73, and the value of the control action is *λ*_0_[*t*_4_] = 0.81. The steady state marking reached is (1.93,4.35,2.73). It is important to note that the target marking (1,4,4) cannot be an achievable steady state marking with the proposed single control action.

All the trajectories in this paper have been obtained by the tool fnyzer (https://bitbucket.org/Julvez/fnyzer.git). This tool makes use of the modeling language Pyomo (Hart et al. 2017; Hart et al. 2011) and solvers, such as Gurobi (Gurobi Optimization 2015) and CPLEX (IBM ILOG CPLEX Optimizer 2010), to solve the programming problems associated with the FNs. The CPU time (Intel i7, 2.00 GHz, 8 GiB, Ubuntu 14.04 LTS) to solve one step of the MPC approach for the FNs in Figs. 6 and 10 was 1.81*s* and 5.93*s* respectively. The CPU time to solve the only programming problem associated with Fig. 8 was 1.27*s*.

## Conclusions

FNs consist of two nets, an event net and an intensity net, that make an explicit distinction between the parts of the system involved in updating the marking in places, i.e. the event net, and the parts of the system involved in the determination of the speeds of transitions, i.e. the intensity net. Both the event and the intensity net are tripartite graphs in which places and transitions are connected by event and intensity handlers, respectively. This way, handlers act as an intermediate layer between places and transitions, which results in a significant modeling power. For instance, a transition in an event net can consume tokens from different sets of places, and a place in an intensity net can regulate the speed of different transitions. The tripartite net structure of event and intensity nets has demonstrated to be useful to model partial observability and resource allocation.

Different types of system uncertainties can be accommodated by FNs through sets of linear inequalities associated with places, transitions, event handlers and intensity handlers. Namely, these inequalities allow the modeling of uncertainty in: a) the initial marking (8); b) the default intensities (15); c) the marking change produced by the execution of actions (4); and d) the intensity change produced by the active tokens (13). FNs account for the potential system trajectories arising as a result of uncertainties by means of a set of constraints that represent necessary reachability conditions. The combination of these constraints with an objective function can be used to obtain a system trajectory that optimizes a given criterion. This approach was successfully used to compute trajectory bounds, for instance, in the presented linear system with uncertain parameters, or to obtain a control law in a system whose control action is modeled by a transition with uncertain default intensity.
